# Cytoplasmic versus nuclear THR alpha expression determines survival of ovarian cancer patients

**DOI:** 10.1007/s00432-020-03241-7

**Published:** 2020-06-12

**Authors:** Nina Ditsch, Sabine Heublein, Udo Jeschke, Cornelia Sattler, Christina Kuhn, Anna Hester, Bastian Czogalla, Fabian Trillsch, Sven Mahner, Jutta Engel, Doris Mayr, Elisa Schmoeckel

**Affiliations:** 1 Department of Obstetrics and Gynecology, LMU Munich, University Hospital, Marchioninistr. 15, 81377 Munich, Germany; 2 Department of Obstetrics and Gynecology, Heidelberg University Hospital, Ruprecht-Karls-University of Heidelberg, 69120 Heidelberg, Germany; 3 Munich Cancer Registry (MCR), Bavarian Cancer Registry—Regional Centre Munich (LGL), Institute for Medical Information Processing, Biometry and Epidemiology (IBE), Ludwig-Maximilians-University (LMU), Munich, Germany; 4 Department of Pathology, LMU Munich, Thalkirchner Str. 36, 80337 Munich, Germany; 5 Department of Obstetrics and Gynecology, University Hospital Augsburg, Stenglinstrasse 2, 86156 Augsburg, Germany

**Keywords:** Thyroid hormone receptor, Ovarian cancer, Overall survival, Nuclear versus cytoplasmic

## Abstract

**Purpose:**

Thyroid hormone receptors (THR) have manifold functions and are involved in the carcinogenesis of several tumor types. Within this study, we aimed to investigate the expression pattern (nuclear versus cytoplasmic) of the THR alpha and its impact on patients survival in ovarian cancer (OvCa).

**Methods:**

The presence of the thyroid hormone receptors THRα, THRα1 and − 2 was investigated in 156 ovarian cancer samples using immunohistochemistry (IHC) using semi-quantitative immunoreactivity (IR) scores and correlated with clinical, pathological data, subtype of ovarian cancer, clinical data, staining of 20 already described OvCa marker proteins and overall survival (OS).

**Results:**

Among all subtypes of OvCa, clear cell carcinomas showed the highest THRα expression. Furthermore, nuclear THRα was associated with a reduced survival in this subtype. However, nuclear expressed THRα1 turned out to be a positive prognosticator for all subtypes of OvCa patients. Nuclear THRα2 is a positive prognosticator for OvCa patients of the serous subtype. In contrast, cytoplasmic expression THRα2 was associated with a reduced OS in all subtypes of OvCa patients; while, cytoplasmic expression of THRα1 is associated with reduced OS in mucinous OvCa patients only. In addition, THRα expression correlates with gonadotropin receptors, steroid hormone receptors, TA-MUC1 and glycodelin.

**Conclusion:**

Depending on nuclear or cytoplasmic expression, our study shows that THRα and its isoforms 1 and 2 provide different prognostic information for ovarian cancer patients. Further investigations should analyze if THRs may represent new endocrine targets for the treatment of ovarian cancer.

**Electronic supplementary material:**

The online version of this article (10.1007/s00432-020-03241-7) contains supplementary material, which is available to authorized users.

## Background

Thyroid-stimulating hormone (TSH) regulates thyroid function by binding to its receptor (thyroid hormone receptor—THR) expressed at the surface of thyroid cells. Recently, it has been demonstrated that THR is abundantly expressed in several tissues apart from the thyroid, among them the normal ovarian surface epithelium. The hormone dependency of the ovaries and the functional similarity of THRs and estrogen- (ER) and progesterone receptors (PR; both act in the nucleus as transcription factors) lead to the hypothesis that THRs may be a prognostic marker in ovarian cancer patients as demonstrated recently for breast cancer patients (Li et al. 2002; Rasmusson et al. 1987; Turken et al. 2003; Ditsch et al. 2013).

The nuclear receptors of thyroid hormones regulate the expression of specific cellular genes by interacting with distinct DNA sequences. They are ligand-activated transcription factors, which regulate the transcription of target genes. THRs are encoded by two genes—THR alpha and beta—located on human chromosomes 17 and 3 (Silva et al. 2002). They have three major isoforms: THRα1, THRα2 and THRβ1 (Ling et al. 2010) with high homology in amino acid composition. The most diversified region between THRα and THRβ is located in the N-terminal area, related to their trans-activation activity (Lazar 1993). Recent studies discovered by oligonucleotide microarray transcriptional profiling that THRα and THRβ mRNAs are among the most strongly expressed nuclear hormone receptor genes in cultured human ovarian surface epithelial (OSE) cells (Rae et al. 2004). The presence of THRα1, ΤHRα2, and THRβ1 transcripts in cultured OSE cells is confirmed and the presence of THRα and THRβ proteins in the OSE cell layer has been demonstrated. Although, THRα and β isoforms are encoded by separate genes, differential promoter usage gives rise to two different THRα receptors, THRα1 and THRα2 (Zhang and Lazar 2000). Unlike THRα1 and THRβ1, which are conventional ligand-activated receptors, THRα2 is a ligand-independent negative regulator of active THRs. Thus, the presence of different THR isoforms, in conjunction with the potential for pre-receptor metabolism of thyroid hormones through expression of activating and inactivating deiodinase enzymes, strengthens the likelihood that the OSE is a physiologically important thyroid hormone target tissue (Rae et al. 2004).

Ovulation is a recurrent inflammatory reaction causing regular and frequent local injury to the ovarian surface during follicular rupture (Espey 1994; Rae and Hillier 2005). Ovarian cancer develops when a mutation or genetic change—spossibly caused by repeat episodes of inflammation-associated DNA damage (Murdoch 1998; Murdoch et al. 1999; Beachy et al. 2004)—occurs in the cells on the surface of the ovaries or in the fallopian tubes and leads to uncontrolled cell growth that may often metastasize (Rasool et al. 2014). Suppression of ovulation by e.g. pregnancy, breast feeding, or oral contraception reduces the risk of ovarian cancer, whereas diseases such as endometriosis, ovarian cysts, and hyperthyroidism are associated with increased risk (Ness and Cottreau 1999; Ness et al. 2000).

Ovarian cancer consists of four histopathological subtypes, represents the fourth most frequent type of cancer among females, and is the leading cause of death from gynecological cancer in the western world. Besides the histopathological subtype, grading, clinical staging and the amount of residual tumor, a number of several putative prognostic markers had been suggested for monitoring this disease (Ditsch et al. 2013). As ovarian cancer is also a thyroid hormone-dependent neoplasm (Shinderman-Maman et al. 2016), T3 has been shown to directly exert inflammatory effects on ovarian surface epithelial cell function in vitro and activate expression of genes associated with inflammation (Cohen et al. 2014; Rae et al. 2007). Studies also indicate that T3 increases the expression of ERα, which strongly associates with the development of epithelial ovarian cancer, which may explain the epidemiological linkage between hyperthyroidism and ovarian cancer (Rae et al. 2007).

The current study examines possible alterations of THR expression in ovarian carcinomas and its implication in ovarian cancer survival. Little is known about the context of thyroid function in ovarian carcinogenesis and the role of THR expression outside the thyroid is not completely understood. From our knowledge of therapy modalities, anti-hormonal therapy like tamoxifen, which unfold its effect via steroid hormone receptors, can be affective in ER-positive ovarian cancers. First in this field, our examinations focuses on the prognostic impact of thyroid hormone receptors of the alpha subtype (general alpha, alpha-1 and alpha-2, respectively) on pathological different ovarian cancer tissues.

## Methods

### Tissue samples

Tissue samples were obtained from 156 patients undergoing gynecological surgery for epithelial ovarian cancer (EOC) at the Department of Obstetrics and Gynaecology of the Ludwig-Maximilians-University Munich. The clinico-pathological parameters are shown in Table 1. Experienced gynaecologic pathologists performed histopathological staining and evaluation according to the criteria of the International Federation of Gynaecologists and Obstetricians (FIGO) and the World Health Organization (WHO). Full slides were used for immuohistochemical stainings. EOC specimens were available in different histological subtypes: serous (*n* = 110) thereof 84 high-grade and 26 low-grade cases, clear cell (*n* = 12), endometrioid (*n* = 21), mucinous (*n* = 13). Patients with ovarian low malignant potential tumors (e.g., Borderline tumors) were excluded from the study and no patients had neo-adjuvant chemotherapy. Patient’s clinical data were available from patient charts, aftercare files and tumor registry database information. The main outcomes assessed were disease recurrence and patient survival. For survival analysis, survival time was defined as the time between the date of primary ovarian cancer diagnosis and the date of death.

**Table 1:** Clinico-pathological parameters of the study group (*n* = 156)

Histological subtype		FIGO stage		Nodal status		Age (years)	
High-grade serous	84 (54%)	I	35 (23%)	N0	56 (36%)	mean	62
Low-grade serous	26 (17%)	II	10 (6%)	N1	54 (35%)	min	33
Endometrioid	21 (13%)	III	107 (69%)	NX	46 (29%)	max	88
Mucinous	13 (8%)	IV	4 (2%)				
Clear cell	12 (8%)						

### Immunohistochemistry

Our group has extensively described immunohistochemistry of THRα, THRα1 and THRα2 on FFPE sections (Ditsch et al. 2012a, 2013). In brief, rabbit polyclonal antibodies detecting THRα (Abcam, Cambridge, UK); Zytomed, Berlin, Germany), THRα1 (Zytomed) and THRα2 (Zytomed)) were stained by employing commercially available kits (Vectastain Elite rabbit-IgG-Kit (VectorLabs, Burlingame, CA); ZytoChem Plus HRP Polymer System (Zytomed). Reference sources for the used antibodies are listed in the Supplementary Table. Appropriate positive (struma, colon and placental tissue) and negative controls were included in each experiment (Supplementary Figure). Tissue sections treated with pre-immune IgGs (supersensitive rabbit negative control, BioGenex, Fremont, CA) instead of the primary antibody served as negative controls. Immunoreactivity was quantified by applying a well-established semi-quantitative scoring system (IR-score; also known as Remmele’s score) by two independent observers (gynecologic pathologists (E.S. and D.M.)) by consensus. This scoring method has already been used in numerous studies (Ditsch et al. 2012b, c; Lenhard et al. 2011, 2012b) of our group. The IRS quantifies immunoreactivity by multiplication of optical staining intensity (graded as 0: no, 1: weak, 2: moderate and 3: strong staining) and the percentage of positive stained cells (0: no staining, 1: ≤ 10% of the cells, 2: 11–50% of the cells, 3: 51–80% of the cells and 4: ≥ 81% of the cells). According to the previously published data, tissue samples that had been assigned an IRS higher than 1 were scored as positive (Lenhard et al. 2012a).

### Statistical analysis methods

The IBM statistic package SPSS (version 25) was used to test data for statistical significance. Differences in THR expression among three or more groups were tested using the non-parametric Kruskal–Wallis rank-sum test and for pairwise comparisons using the nonparametric Mann–Whitney U rank-sum test. Correlation analysis was performed using the Spearman correlation coefficient. Overall survival (years) was compared by Kaplan–Meier graphics and differences in patient overall survival times were tested for significance using the chi-square statistics of the log rank test. For multivariate analyses, the cox regression model for overall survival was used. Data were assumed to be statistically different in case of *p* < 0.05.

## Results

### THRα expression according to EOC subtypes

**Fig. 1:**
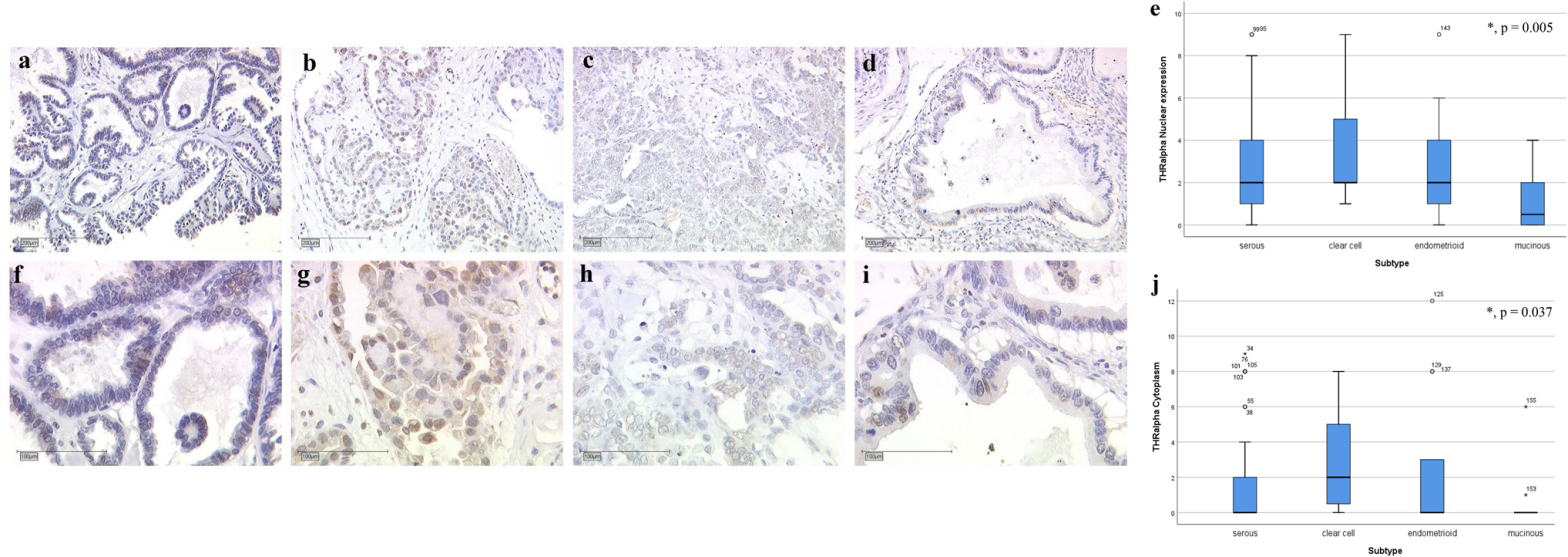
**a** THRα expression in serous carcinoma (10 × lens). **b** THRα expression in clear cell carcinoma (10 × lens). **c** THRα expression in endometrioid carcinoma (10 × lens). **d** THRα expression in mucinous carcinoma (10 × lens). **e** Summary of THRα expression in different carcinoma subtypes (nuclear expression). **f** THRα expression in serous carcinoma (25 × lens). **g** THRα expression in clear cell carcinoma (25 × lens). **h** THRα expression in endometrioid carcinoma (25 × lens). **i** THRα expression in mucinous carcinoma (25 × lens). **j** Summary of THRα expression in different carcinoma subtypes (cytoplasmic expression)

THRα expression showed significant differences within the histological subtype, accounting nuclear as well as cytoplasmic staining. Serous carcinomas showed only faint expression of THRα in the nucleus (median IRS = 2) as well as in the cytoplasm (median IRS = 0; Fig. 1a = 10 × lens, Fig. 1f = 25 × lens). A more intense staining was observed in the clear cell cases in the nucleus (median IRS = 2) as well as in the cytoplasm (median IRS = 2; Fig. 1b = 10 × lens, Fig. 1g = 25 × lens). The endometrioid subtype showed similar expression schemas as the serous subtype in the nucleus (median IRS = 2) as well as in the cytoplasm (median IRS = 0; Fig. 1c = 10 × lens, Fig. 1h = 25 × lens). The lowest expression of THRα was found in the mucinous subtype in the nucleus (median IRS = 1) as well as in the cytoplasm (median IRS = 0; Fig. 1d = 10 × lens, Fig. 1i = 25 × lens). A summary of the staining results is shown in Fig. 1e for the nuclear staining (*p* = 0.005) and Fig. 1j for the cytoplasmic staining (*p* = 0.037).

THRα1 as well as THRα2 showed no significant different expression according to the histological subtype. The median expression of THRα1 in the nucleus was 2 and the median expression in the cytoplasm was 0. The median expression of THRα2 in the nucleus was 6 and, therefore, much more intense compared to THRα and − α1, respectively. The median expression of THRα2 in the cytoplasm was 0. There was no significant different expression of the three THRα subtypes according to grading, FIGO staging or age at surgery.

### Correlation analyses

Using recently published data by our institute, we were able to correlate the expression of all THRα subtypes stained with former investigation results. There are significant correlations with the gonadotropin receptors (Lenhard et al. 2011) and the luteinizing hormone (LH)-receptor ligand hCG (Lenhard et al. 2012b); specifically, THRα staining in the nucleus showed a positive correlation to the follicle stimulating hormone receptor (FSHR) (correlation coefficient (cc) = 0.181; *p* = 0.027) and a negative correlation to hCG (cc = − 0.247, *p* = 0.003). In opposite, THRα in the cytoplasm showed a positive correlation with the luteinizing hormone/choriogonadotropin receptor (LH/hCGR) (cc = 0.199, *p* = 0.014) and a positive correlation to hCG (cc = 0.187, *p* = 0.027). The THRα1 expression in the cytoplasm is positively correlated with hCG (cc = 0.278, *p* = 0.001). THRα2 in the nucleus showed a positive correlation to FSHR (cc = 0.185, *p* = 0.024). In addition, there are also positive correlations with the classical steroid hormone receptors, which were analyzed by our research group too (Lenhard et al. 2012a). THRα staining in the nucleus showed a positive correlation with the ERβ (cc = 0.213, *p* = 0.009) and with the PRα (cc = 0.172, *p* = 0.035). The THRα1 expression in the cytoplasm is positively correlated with ERβ (cc = 0.219, *p* = 0.006). THRα2 in the nucleus showed positive correlation with ERα (cc = 0.247, *p* = 0.002) and with PRα (cc = 0.219, *p* = 0.007). In addition to the classical estrogen receptors, also the GPER (Heublein et al. 2014; Heublein et al. 2013a, b) showed positive correlation with THRα staining in the nucleus (cc = 0.219, *p* = 0.007) and with THRα2 in the nucleus (cc = 0.252, *p* = 0.002).

Another positive correlation was found within the tumor-associated mucin 1 epitop (TA-MUC1) detected with the Gatipotuzumab antibody formerly known as PankoMab (Dian et al. 2013; Jeschke et al. 2012) and THRα staining in the nucleus (cc = 0.279, *p* = 0.001). In contrast, THRα1 expression in the cytoplasm is negatively correlated with TA-MUC1 (cc = -0.195, *p* = 0.019). TA-MUC1 as membrane bound protein can also be translocated to the cytoplasm of tumor cells (Heublein et al. 2015). In that case, it is negatively correlated with the expression of THRα1 in the nucleus (cc = − 0.166, *p* = 0.048) and THRα2 in the nucleus (cc = − 0.268, *p* = 0.001). An immunosuppressive glycoprotein that is connected to TA-MUC1 is glycodelin and its specific glycoform glycodelin A (Lenhard et al. 2013; Scholz et al. 2012). Glycodelin A showed a positive correlation with THRα2 in the cytoplasm (cc = 0.170, *p* = 0.037). Glycodelin showed positive correlation with THRα in the nucleus (cc = 0.241, *p* = 0.003) as well as in the cytoplasm (cc = 0.231, *p* = 0.004). THRα2 expression in the nucleus is positively correlated with glycodelin (cc = 0.265, *p* = 0.001).

### Survival analyses

**Fig. 2:**
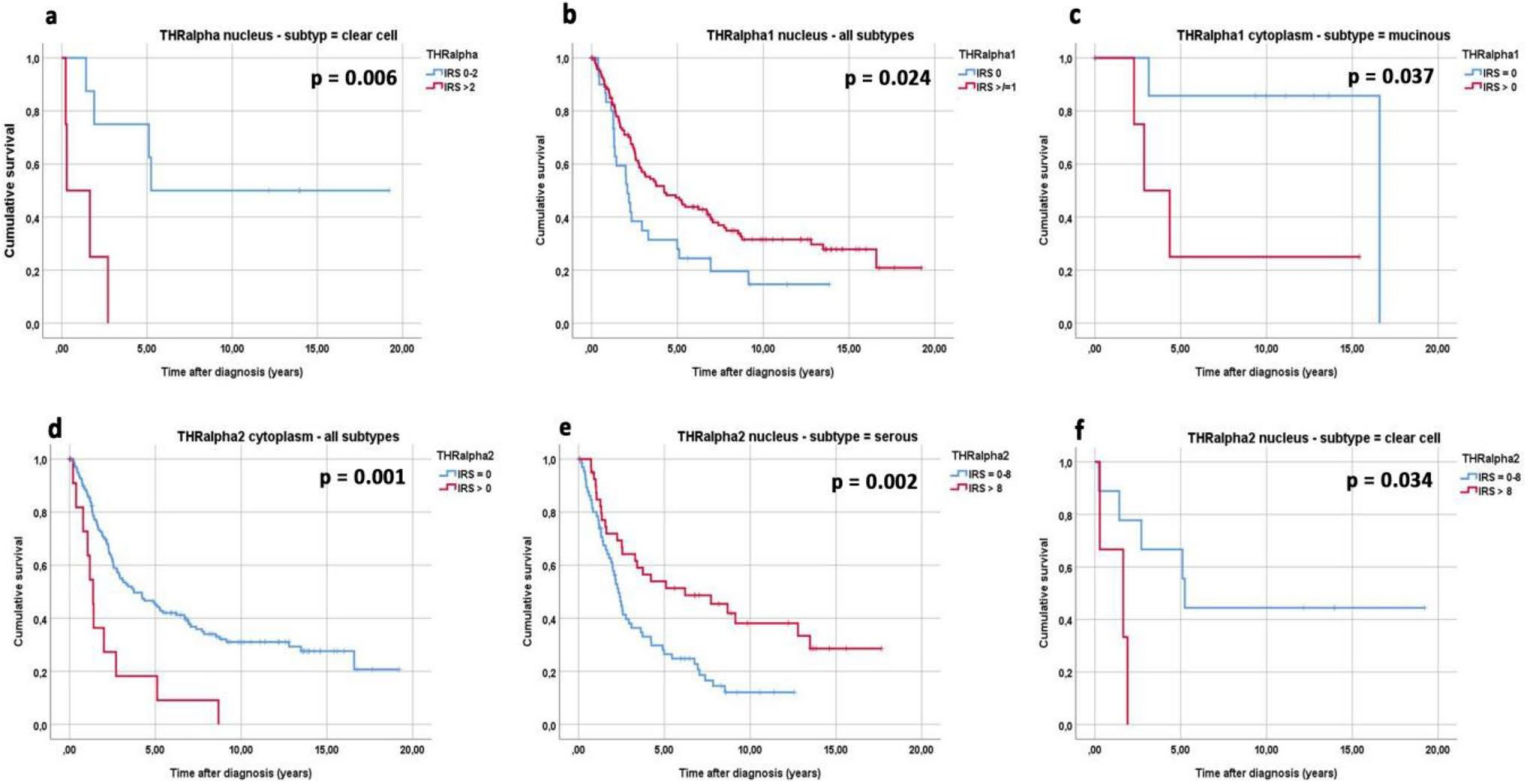
Kaplan–Meier estimates of THRα expression, THRα1 expression and THRα2 expression were analyzed. In the clear cell subtype, patients with a high nuclear expression of THRα showed a significantly reduced overall survival compared with patients with a low nuclear expression (**a**). In addition, high nuclear THRα1 expression was associated with significantly better overall survival in all ovarian cancer subtypes compared to patients with a low nuclear THRα1 expression (**b**). Patients with high THRα1 expression in the cytoplasm and mucinous subtype had a significantly decreased overall survival compared with those mucinous carcinoma patients with low cytoplasmic expression (**c**). High cytoplasmic THRα2 expression was associated with a significantly reduced overall survival in all ovarian cancer subtypes compared to patients with a low cytoplasmic THRα2 expression (**d**). In the serous subtype, patients with a high nuclear expression of THRα2 showed a significantly better overall survival compared with patients with a low nuclear expression (**e**). Finally, in the clear cell subtype, patients with a high nuclear expression of THRα showed a significantly reduced very low overall survival (all patients deceased within two years) compared to patients with a low nuclear expression (**f**)

The expression of the general THRα is connected to significantly reduced overall survival in the subgroup of clear cell carcinomas. The median survival for THRα-negative patients is 5.24 years in contrast to only 0.29 years for patients showing THRα expression in the nucleus (Fig. 2a, *p* = 0.006).

The THRα isoforms – α1 and – α2 are in general positive prognosticators if expressed in the nucleus and negative prognosticator if expressed in the cytoplasm, respectively. In detail, THRα1 is a general positive prognosticator if expressed in the nucleus with a median survival of 4.22 years for patients positive for THRα1 and 2.08 years for patients that do not express THRα1 in the nucleus (Fig. 2b, *p* = 0.024). Subgroup analyses of mucinous carcinomas showed that THRα1 is a negative prognosticator if expressed in the cytoplasm. The median survival time is 16.59 years for mucinous carcinoma patients that do not express THRα1 in the cytoplasm and 2.87 years for mucinous carcinoma patients with cytoplasmic THRα1 expression (Fig. 2c, *p* = 0.037).

The THRα2 receptor in general is a negative prognosticator if expressed in the cytoplasm. The median survival time is 3.75 years for patients and 1.37 years for patients with THRα2 in the cytoplasm (Fig. 2d, *p* = 0.001). Nuclear expression of THRα2 is not a general positive prognosticator. This can be found in the subgroup of serous high-grade carcinomas. The mean survival time for high-grade serous carcinoma patients with nuclear THRα2 expression is 6.21 years in contrast to 2.32 years for patients with no nuclear THRα2 expression (Fig. 2e, *p* = 0.002). It is remarkable that patients with clear cell carcinomas show opposite results. The median survival time for clear cell carcinoma patients with nuclear THRα2 expression is only 1.65 years in contrast to 5.24 years for patients with no nuclear THRα2 expression (Fig. 2f, *p* = 0.034).

The results of the survival analyses in correlation with the histological subtype and staining localization of THRα, THRα1 and THRα2 are summarized in Table 2.

**Table 2:** Results of the survival analyses in correlation to the histological subtype and staining localization of THRα, THRα1 and THRα2

Histological subtype	THRα IRS > 0	THRα1 IRS > 0		THRα2 IRS > 0	
	Nucleus	Nucleus	Cytoplasm	Nucleus	Cytoplasm
Total (*n* = 156)	n.s	pos. pro. *p* = 0.024	n.s	n.s	neg. pro *p* = 0.001
High-grade serous (*n* = 84)	n.s	n.s	n.s	pos. pro *p* = 0.002	n.s
Low-grade serous (*n* = 26)	n.s	n.s	n.s	n.s	n.s
Endometrioid (*n* = 21)	n.s	n.s	n.s	n.s	n.s
Mucinous (*n* = 13)	n.s	n.s	neg. pro *p* = 0.037	n.s	n.s
Clear cell (*n* = 12)	neg. pro *p* = 0.006	n.s	n.s	neg. pro *p* = 0.034	n.s

*n.s.* not significant; *pos. pro.*  positive prognosticator; *neg. pro.* negative prognosticator

### Comparison of THRα, − α1 and − 2 expression in low-grade and high-grade serous ovarian cancer

**Fig. 3:**
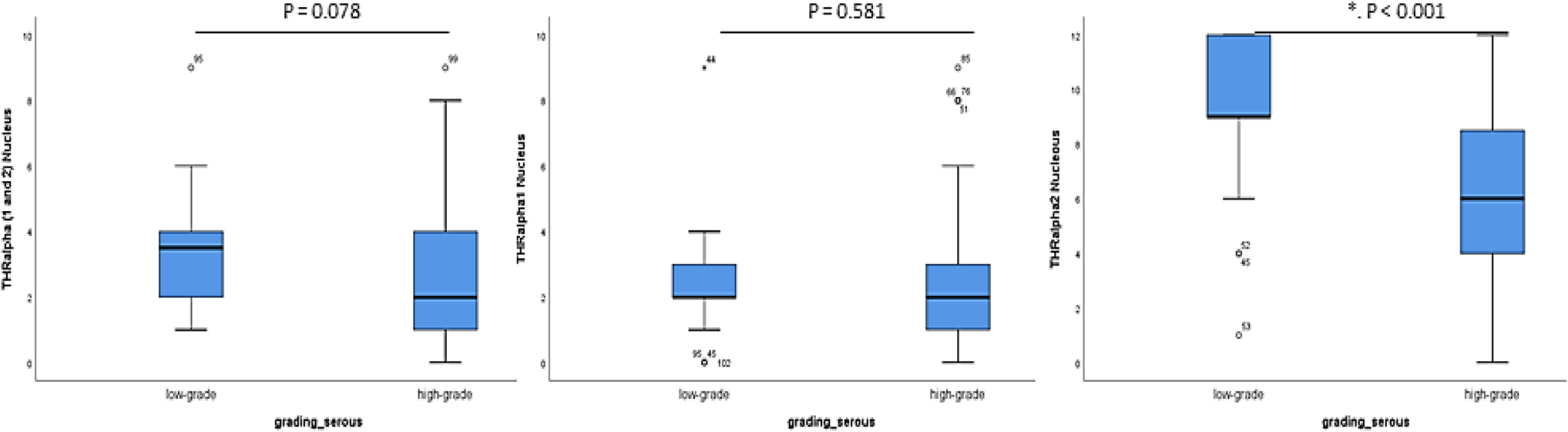
Comparison of immunohistochemical staining results of the different THR (median values) in the nucleus of the high- and low-grade serous ovarian cancer subtypes. (*IRS* Immunoreactive Score, *THR*: Thyroid Receptor). The expression of THRα2 in the nucleus is significantly different in low-grade compared to high-grade serous carcinomas (marked by an asterisk)

As shown in Fig. 3, the expression of all three α-subunits is higher in the nucleus of low-grade serous ovarian cancer cases with a trend to significance in the general THRα (*p* = 0.078), no significance for THRα1 and a significantly higher THRα2 expression in low-grade serous cancer cases compared to high-grade subtype (the receiver operating characteristic curve (ROC) analyses were performed to calculate the optimal cut-off values between low and high expression of the different THRs).

### Cox regression analyses of survival

Cox regression was performed to identify independent predictors for OS. Pattern of age at surgery failed to remain significant within multivariate testing; while grading, FIGO staging, THRα1 in the nucleus (Table 3A, *p* = 0.043) and THRα2 in the cytoplasm (Table 3B, *p* = 0.002) were still predictive in multivariate testing sets regarding all subtypes of the study group. Due to missing clinical data in single cases, cox regression analyses were available in 146 out of 156 cases.

**Table 3:** Multivariate survival analyses with the overall survival time for (A) THRα1 expression in the nucleus, (B) THRα2 expression in the cytoplasm, regarding patients age, histological subtype, grading, and staging (*n* = 146)

Variables	*p* value	Hazard ratio	95.0% Confidence Interval	
			Lower	Upper
(A) THRα1 (nucleus)				
IRS > 0 versus IRS 0	0.049	0.618	0.383	0.997
FIGO				
I/II versus III/IV	0.001	2.761	1.510	
Grading				
G1/low grade versus	0.002	2.753	1.457	5.199
G2/3/high grade				
Histological subtype				
all subtypes versus high-grade serous	0.964	0.994	0.783	1.263
Age				
< 60 versus ≥ 60 years	0.116	0.717	0.473	1.085
(B) THRα2 (cytoplasm)				
IRS > 0 versus IRS 0	0.002	2.790	1.466	5.310
Age				
< 60 versus ≥ 60 years	0.212	0.769	0.509	1.161
Histological subtype				
all other subtypes versus high-grade serous	0.673	0.950	0.747	1.207
Grading				
G1/low-grade versus	0.001	0.325	0.171	0.618
G2/3/high-grade				
FIGO				
I/II versus III/IV	0.001	0.365	0.201	0.662

## Discussion

Within this study, we analysed the prognostic value of the thyroid hormone receptor alpha forms 1 and 2. The general THRα has prognostic value only in clear cell carcinomas, where it is expressed at the highest immune scores. The differential analyses of nuclear versus cytoplasmic expression of THRα1 and THRα2 revealed striking differences concerning the overall survival of ovarian cancer patients. The thyroid hormone receptor alpha (THRα) exhibits a dual role as an activator or repressor of gene transcription. Former studies showed that THRα, formerly thought to reside solely in the nucleus and tightly bound to the DNA, shuttles rapidly between the nucleus and the cytoplasm (Bunn et al. 2001; Maruvada et al. 2003).

The role of thyroid hormones and its receptors was not very well understood in ovarian cancer biology for a longer time, only very recent publication showed their tremendous roles for this deadly disease.

Early investigations with ovarian cancer cell lines and T3, T4 and reversed T3 stimulation did not result in sufficient stimulation or inhibition outcomes (Martinez et al. 2000). Later, it was found that messenger RNA transcripts for THRα1, THRα2, T3 activating deiodinase 2 and inactivating deiodinase 3 are present in primary ovarian surface epithelial cell cultures (Rae et al. 2007). A more recent study described that for ovarian cancer patients, conflicting results were observed for T3 and T4 levels in the serum. Insignificant differences were found for T3 (*p* = 0.209) and T4 (*p* = 0.050) as compared to controls (Rasool et al. 2014).

An actual study described that αvβ3 integrin, a plasma membrane receptor that binds the thyroid hormones T3 and T4, is overexpressed in ovarian cancer (Shinderman-Maman et al. 2016). Both hormones induced cell proliferation and significantly reduced the expression of genes that inhibit cell cycle particularly in ovarian cancer cells (OVCAR-3) with high integrin expression (Shinderman-Maman et al. 2016). The same group studied the expression of fifteen genes involved in DNA repair, cell cycle, apoptosis, and tumor suppression in OVCAR-3 and A2780 cell lines, using real-time PCR following short incubation with T3 or T4 (Shinderman-Maman et al. 2018). The thyroid hormones downregulated the expression of the majority of genes examined, showing that these hormones influence the expression of cancer-relevant genes in ovarian cancer (Shinderman-Maman et al. 2018). The same group hypothesized that natural thyroid hormone derivatives may antagonize these actions. The three antagonists, tetraiodoacetic acid (tetrac), triiodothyroacetic acid (triac) and 3-iodothyronamine (T1AM) inhibited cell proliferation and induced cell death and DNA damage in the two ovarian cancer cell lines (OVCAR3 and A2780). Therefore, they concluded that the cytotoxic potential of thyroid hormone derivatives, tetrac, triac and T1AM, in ovarian cancer might provide a much-needed novel therapeutic approach (Shinderman-Maman et al. 2017).

Based on the results of the former study, another group described that thyroid hormone causes elevated phosphorylation and nuclear enrichment of ERα (Hsieh et al. 2017). In addition, confocal microscopy indicated that both T4 and estradiol caused nuclear translocation of integrin αv and phosphorylation of ERα (Hsieh et al. 2017). Within our study, we found a positive correlation between the THRα2 in the nucleus and ERα. We also found positive correlation of THRα in the nucleus and ERβ, assuming that thyroid hormones not only elevate the nuclear enrichment of ERα but also might influence ERβ. However, our correlations referred to the whole study cohort and did not focus on the histological subtypes. Another study showed that THRα1 inhibits the ERα transactivation from the consensus estrogen response element (ERE). In contrast, the ligand bound THRβ1 facilitates ERβ-mediated transactivation (Vasudevan et al. 2001). We also found a positive correlation between the GPER and THRα. Sheng et al. showed that the GPER together with integrin αvβ3 participate in the induction of male germ cell proliferation and thyroid transcription disruption after low-dose Bisphenol A treatment (Sheng et al. 2019). Another correlation of our study was found between THRα in the nucleus and the FSH receptor; whereas, the THRα expression in the cytoplasm showed a positive correlation to the LH/hCG receptor. It has been known for a longer time that LH, FSH, and TSH show low-level cross-reactivity between their respective receptors (Tonacchera et al. 2006). Vissenberg et al. explained that T3 in combination with FSH enhances granulosa cell proliferation and inhibits granulosa cell apoptosis by the PI3K/Akt pathway (Vissenberg et al. 2015). They also described that T3 is considered a biological amplifier of the stimulatory action of gonadotrophins on granulosa cell function (Vissenberg et al. 2015). Because the exclusive expression of the FSHR has already been described by our group as a negative prognosticator in ovarian cancer cases, our finding about enhanced expression of both FSHR and THRα in the nucleus might lead to new treatment strategies for this type of cancer (Lenhard et al. 2011). This assumption might also apply for the antibody Gatipotuzumab and its TA-MUC1 epitope (Heublein et al. 2019), which showed an inverse correlation to THRα1 and -2 expression either in the nucleus or in the cytoplasm, respectively.

In addition, T4 has been shown to promote ovarian cancer cell proliferation via integrin αvβ3. T4 also induced the activation of ERK1/2 and expression of programmed death-ligand 1 (PD-L1) in ovarian cancer cells (Chin et al. 2018). In contrast, resveratrol binds to integrin αvβ3 at a discrete site and induces p53-dependent anti-proliferation in malignant neoplastic cells. T4 impairs resveratrol-induced anti-proliferation in human ovarian cancer cells and T4 inhibited resveratrol-induced nuclear accumulation of COX-2 (Chin et al. 2018). Furthermore, T4 increased expression and cytoplasmic accumulation of PD-L1, which in turn acted to retain inducible COX-2 in the cytoplasm (Chin et al. 2018). Thus, T4 inhibits COX-2-dependent apoptosis in ovarian cancer cells by retaining inducible COX-2 with PD-L1 in the cytoplasm (Chin et al. 2018).

Recently, the interplay between epithelial–mesenchymal transition (EMT) and the thyroid hormones-αvβ3 axis in ovarian cancer was investigated (Weingarten et al. 2018). It was shown that the transcription of mesenchymal markers, β-catenin, zeb-1, slug/snail, vimentin, and n-cadherin was hardly affected by T3 and T4, while that of the epithelial markers, e-cadherin and zo-1, and was inhibited after treatment with thyroid hormones. These results suggest a novel role for the thyroid hormone-αvβ3 axis in EMT, with possible implications for ovarian cancer metastasis (Weingarten et al. 2018).

Finally, a group investigated the role of the thyroid hormone receptor Interactor 13 (TRIP13) in epithelial ovarian cancer (EOC) (Zhou and Shu, 2019). Bioinformatics analysis showed that TRIP13 was one of the most significantly upregulated proteins in EOC. Results of the described study showed that TRIP13 acted as an onco-promotive regulator in EOC development by modulating the Notch signaling pathway (Zhou and Shu, 2019).

A large demographic study, the “Ovarian Cancer Association Consortium”, showed that hyperthyroidism within the 5 years before ovarian cancer diagnosis was associated with an increased risk of death (Minlikeeva et al. 2017). These very recent results were accompanied by the fact that a more modest association was observed with the history of hypothyroidism (*n* = 624 cases) and mortality (Minlikeeva et al. 2017).

In sum, the results of the experimental and demographic studies about the roles of thyroid hormones, its receptors and interacting proteins. There is growing body of evidence that they play a major role in ovarian cancer biology and survival of ovarian cancer patients. Only recent studies were able to bring new light into this area of research.

## Conclusions

With our study, we could show that there is a direct link between nuclear expression of THRα1 or − 2 and better survival in EOC, except for the subgroup of clear cell carcinomas. The latter group seems to have different properties concerning THRα expression. Shifting the expression of THRα1 or − 2 to the cytoplasm seems to be connected with reduced overall survival in EOC cases. Therefore, the search for THRα interacting factors that prevent this shift to the cytoplasm seems to be a useful new approach for the search of future treatment strategies against the threatening disease of Epithelial Ovarian Cancer.

## Electronic supplementary material

Below is the link to the electronic supplementary material.

Supplementary file1 (DOCX 14 kb)

Supplementary file2 Positive and negative control staining for THRα antibodies used: (a) THRα staining in Struma tissue (10x lens). (b) THRα1 staining in colon tissue (10x lens). (c) THRα2 staining in placental tissue (10x lens). (d) THRα1 negative control in colon tissue (10x lens). (e) THRα2 negative control in placental tissue (10x lens). (f) THRα staining in Struma tissue (25x lens). (g) THRα1 staining in colon tissue (25x lens). (h) THRα2 staining in placental tissue (25x lens). (i) THRα1 negative control in colon tissue (25x lens). (j) THRα2 negative control in placental tissue (25x lens). (PPTX 375 kb)
