# Supplementary material for: Cytoplasmic versus nuclear THR alpha expression determines survival of ovarian cancer patients
Source: J Cancer Res Clin Oncol. 2020 Jun 12;146(8):1923–32. doi: 10.1007/s00432-020-03241-7 (PMC7324415; doi:10.1007/s00432-020-03241-7)
Supplement: Supplementary file 1 — Supplementary file1 (DOCX 14 kb) [file 432_2020_3241_MOESM1_ESM.docx]

**Supplementary Table:** Reference sources for the used antibodies.

| **Antigen** | **Antibody source,**  **catalog number** | **Clone** | **Dilution** | **Detection system** |
| --- | --- | --- | --- | --- |
| THRα | Abcam  ab15543 | Poly-  clonal | 1:400 | Vectastain Elite ABC Kit (Rabbit IgG) |
| THRα1 | Abcam  ab53729 | Poly-clonal | 1:200 | ZytoChem Plus HRP Polymer System |
| THRα2 | Serotec MCA2842 | Mouse IgG1 mono-clonal  Clone: 1330 | 1:200 | ZytoChem Plus HRP Polymer System |
