# Supplementary figures and images for: Cytoplasmic versus nuclear THR alpha expression determines survival of ovarian cancer patients
Source: J Cancer Res Clin Oncol. 2020 Jun 12;146(8):1923–32. doi: 10.1007/s00432-020-03241-7 (PMC7324415; doi:10.1007/s00432-020-03241-7)

## Slide 1
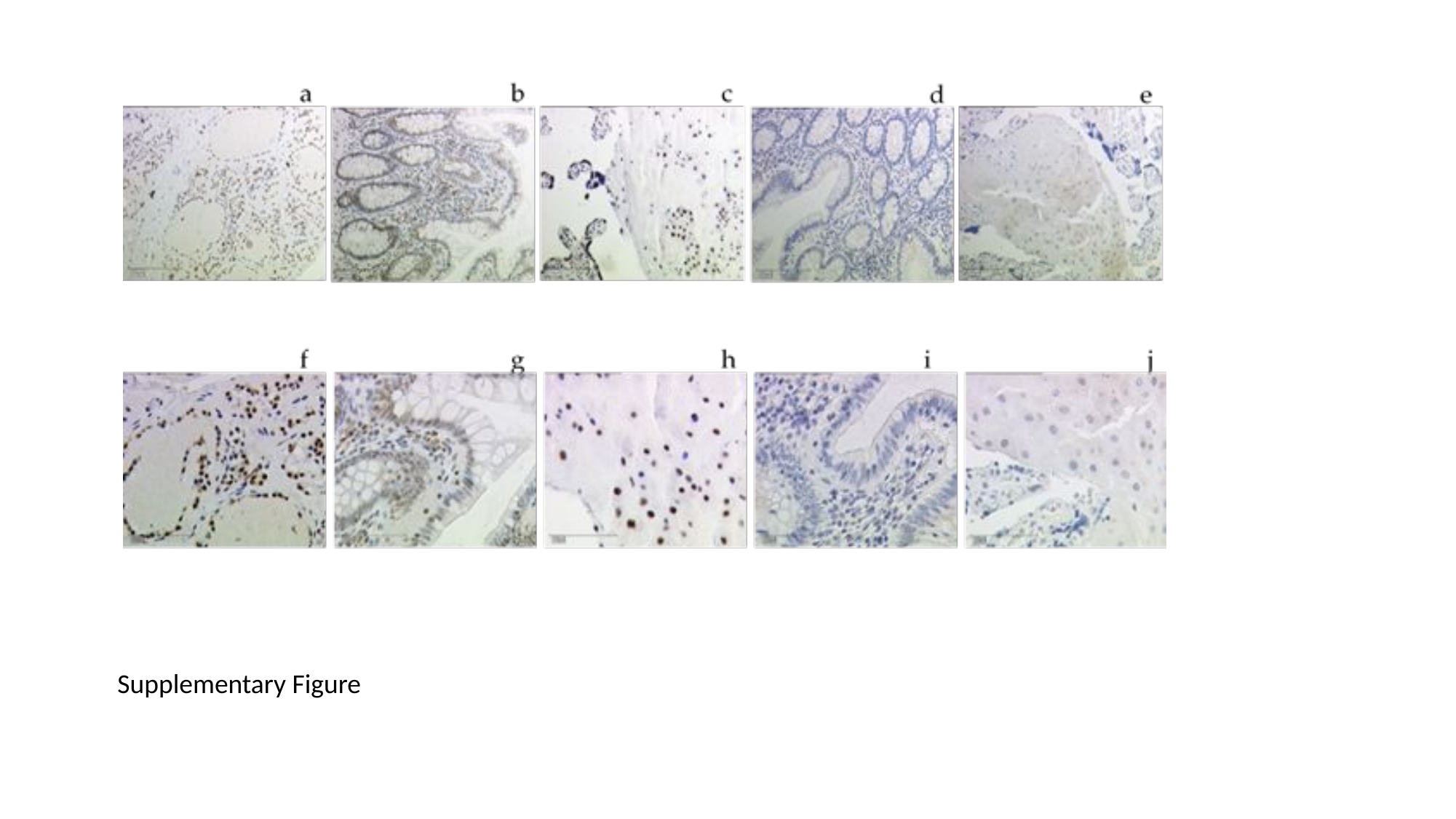

Supplementary Figure

Supplement: Supplementary file 2 — Supplementary file2 Positive and negative control staining for THRα antibodies used: (a) THRα staining in Struma tissue (10x lens). (b) THRα1 staining in colon tissue (10x lens). (c) THRα2 staining in placental tissue (10x lens). (d) THRα1 negative control in colon tissue (10x lens). (e) THRα2 negative control in placental tissue (10x lens). (f) THRα staining in Struma tissue (25x lens). (g) THRα1 staining in colon tissue (25x lens). (h) THRα2 staining in placental tissue (25x lens). (i) THRα1 negative control in colon tissue (25x lens). (j) THRα2 negative control in placental tissue (25x lens). (PPTX 375 kb) [file 432_2020_3241_MOESM2_ESM.pptx]
